# Evidence for Public Policies to Prevent Suicide Death in the United States

**DOI:** 10.1146/annurev-publhealth-071723-121359

**Published:** 2025-01-07

**Authors:** Jonathan Purtle, Amanda I. Mauri, Michael A. Lindsey, Katherine M. Keyes

**Affiliations:** 1 Department of Public Health Policy and Management, School of Global Public Health, New York University, New York, NY, USA; 2 Silver School of Social Work, New York University, New York, NY, USA; 3 Department of Epidemiology, Mailman School of Public Health, Columbia University, New York, NY, USA

**Keywords:** suicide, policy, suicide prevention, policy implementation, United States

## Abstract

Suicide rates have increased in the United States in recent years. Public policies have great potential to prevent suicide death, and well-designed quasi-experimental studies have identified policies that are effective at reducing suicide rates; however, evidence about these policies has not been synthesized. This review summarizes evidence across three domains of public policies: (*a*) policies that affect structural determinants of suicide risk (e.g., policies that improve economic security), (*b*) policies that promote access to clinical services (e.g., Medicaid expansion), and (*c*) policies that limit access to lethal means for completing suicide (e.g., policies that restrict access to firearms). The historical context of suicide prevention in US public policy is provided, considerations for successful suicide prevention policy implementation are discussed—such as policy awareness among key groups, enforcement, and sufficient funding—and priority areas for future research are enumerated.

## INTRODUCTION

Suicide deaths are preventable. An interplay of biological, social, cultural, economic, political, and environmental factors affect the risk of suicide death, and many of these factors can be modified by public policies (e.g., directly by policies that fund the erection of barriers at suicide jumping hot spots and restrict access to other lethal means, and indirectly by policies that provide income supports and reduce chronic stress) (24, 35, 42, 73, 76, 80, 126, 127, 133, 143). Given that public policies often affect entire populations, policies that have even small effects on preventing suicide can save significant numbers of lives. The importance of public policy in suicide prevention is recognized in reviews of the epidemiology and sociology of suicide (6, 77, 141) and multilevel approaches to suicide prevention (53, 73, 80, 98, 126, 143). However, no prior reviews have integrated knowledge about the specific types of public policies that have demonstrated evidence of preventing suicide deaths. This review intends to address this critical and timely gap in the literature.

We first provide brief overviews of the epidemiology of suicide and historical context related to public policy and suicide prevention in the United States. We then delineate the parameters of our review, offer a typology of public policies that may affect suicide death rates, and review evidence of these policies’ effects. We conclude by discussing the importance of policy implementation processes in suicide prevention and articulate important knowledge gaps and key priorities for future research.

### Epidemiology of Suicide in the United States

An estimated 49,449 people died by suicide in the United States in 2022 (31). Suicide is among the top 10 leading causes of death in the United States between the ages of 10 and 64 and among the top five leading causes of death between the ages of 10 and 44 (23). Suicide death rates entered a period of near-record decline in the 1990s and 2000s and then increased by 37% since 2000 and 62% for those aged 10–24 between 2010 and 2020 (22). Firearms are the most common method of suicide death in the United States due to the high lethality of self-inflicted gunshot wounds and the high prevalence of firearms in homes (82, 129).

Suicide is unequally distributed by demographics and geography. While nonfatal attempts are more common among women (84), men die by suicide at considerably higher rates, likely due to the use of more lethal means of suicide attempts. Rates of suicide are highest in American Indian and Alaska Native (AI/AN) populations. In 2021, the rate of suicide among AI/AN populations was 28.1 per 100,000, more than double the national rate, and also increased most rapidly compared with other racial/ethnic groups between 2018 and 2021 (128). By geography, suicide rates are highest in rural regions, including Montana, Wyoming, Utah, and New Mexico (21). Other determinants of suicide death are multifactorial and include occurrences of mental health crises and depressive episodes, isolation and loneliness, illness, and acute stressful life events (42). However, predicting suicide death at the individual level remains challenging (61).

### Historical Context: Origins and Trends in Suicide Prevention Policy in the United States

**Figure 1:**
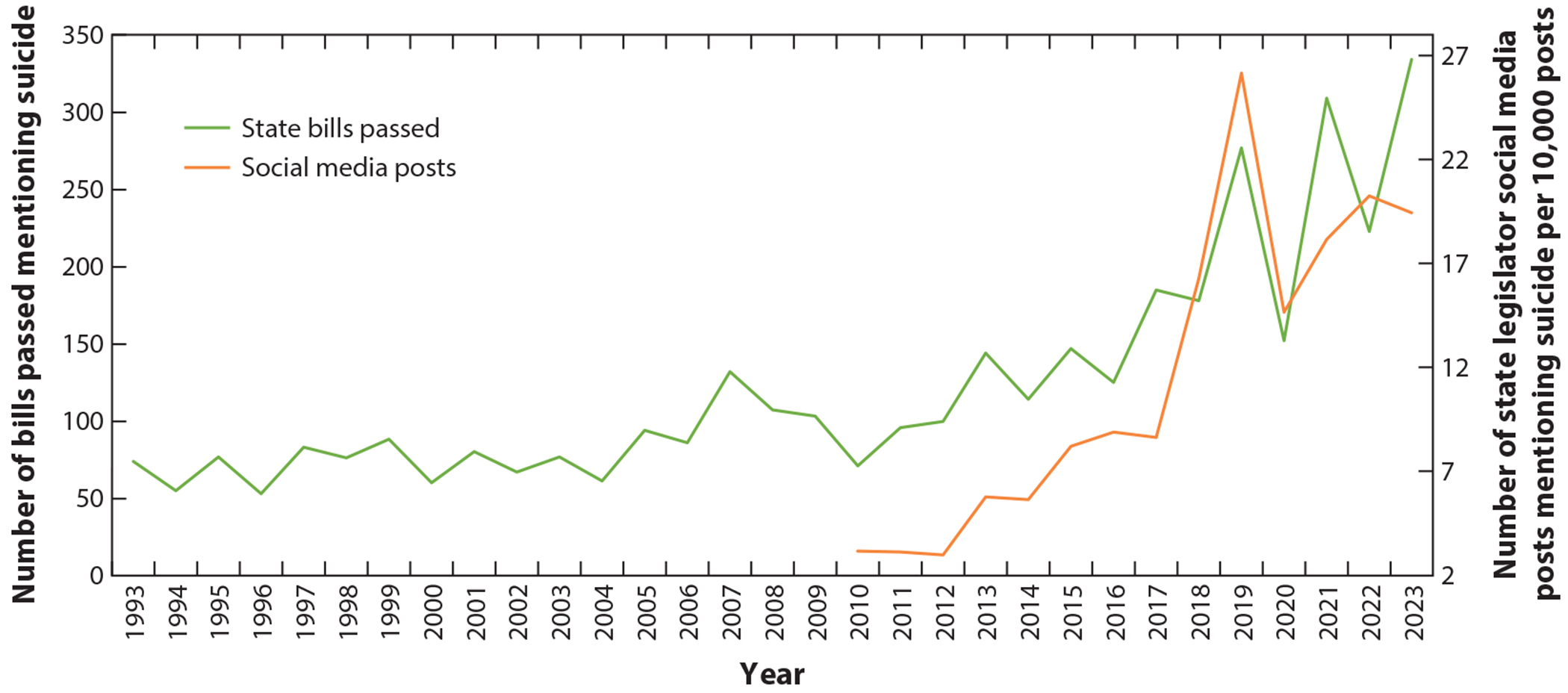
Annual trend in number of state laws passed and rate of state legislator social media posts mentioning suicide. State law data obtained from LexisNexis. Social media post data obtained from Quorum.

As described in Bell’s (10) *We Shall Be No More: Suicide and Self-Government in the Newly United States*, discussions about suicide and the role of collective action, government, and policies in preventing it have existed in the United States since its founding. Before the American Revolution, suicide was viewed as an “English malady”; however, by the late eighteenth century, there was widespread concern that suicide was becoming “a defining feature of life in the United States” (10, p. 3). In response, humane societies of the late eighteenth century developed and implemented early antecedents to modern suicide prevention and crisis interventions, including “The Method.” This approach entailed placing emergency response kits with medicines such as emetics and smelling salts alongside customized grappling devices at locations where suicide attempts often occurred, such as bridges and wharfs. These societies also led public education campaigns about how to perform resuscitation. Leaders of these humane societies included signers of the Declaration of Independence, prominent printers and lawyers, and Revolutionary War officeholders, demonstrating that suicide prevention was a significant concern among political leaders and *de facto* policymakers of the early American republic (10, 132). Nonetheless, the US Congress took an additional century and a half to explicitly incorporate suicide into federal legislation (see the sidebar titled Increasing Concern About Suicide Among Policymakers in the United States; Figure 1).

### Scope of Review and Caveats for Consideration

A wide breadth of factors affect suicide risk (24, 35, 42, 73, 76, 80, 126, 127, 133, 143), and an even broader range of policies affect these factors. Given the potentially boundless influence of policies on suicide, we delineate the boundaries of our review in regard to our outcome of focus, how we operationalized “policy,” and country context. In terms of the outcome, we focus primarily on suicide death as opposed to other, related outcomes such as nonfatal suicide attempts, psychological states such as suicidal ideation or suicide crisis syndrome, or suicide bereavement (63, 97, 120). Although these are important outcomes in and of themselves, the effects of policies on these outcomes may be different than for suicide death. Also, suicide death data are routinely collected and reported across geopolitical jurisdictions (e.g., states) over time. These mortality data allow for jurisdictions with varying policies to be compared and the effects of policies on suicide death to be inferred.

In terms of operationalizing policy, we focus on public policies, such as laws created by elected officials and public administrative regulations created by public agency leaders. We do not consider the effects of private health care system or organizational policies, despite their important role in multilevel suicide prevention strategies (e.g., such as hospital policies that mandate suicide screening). Consistent with definitions of “population-based interventions” (102), of which public policies are a type, we do not consider the effects of individual-level clinical or programmatic interventions or policies that serve primarily to fund individual-level interventions [e.g., the Garrett Lee Smith Memorial Act (Pub. L. 108–355), which has been the focus of prior research (43, 46, 136)].

In terms of country context, we focus primarily on research evaluating the effects of state policies in the United States. We focus on the United States because the federalist, decentralized structure of the country—in which 50 states have broad discretion to adopt different policies at different times—facilitates the use of quasi-experimental designs capable of estimating the independent effects of specific policies on suicide death rates (81).

In terms of caveats, a methodological challenge relates to the fact that suicide death is a relatively infrequent event, and thus policy studies often lack adequate statistical power to identify significant effects. This limited statistical power increases the risk of a type 2 error, in which a study concludes that a policy does not have an effect on suicide death rates when in reality it does. Limited statistical power also constrains the ability to assess policy effect heterogeneity across different demographic groups (25).

## EVIDENCE FOR PUBLIC POLICIES TO PREVENT SUICIDE DEATH

**Figure 2:**
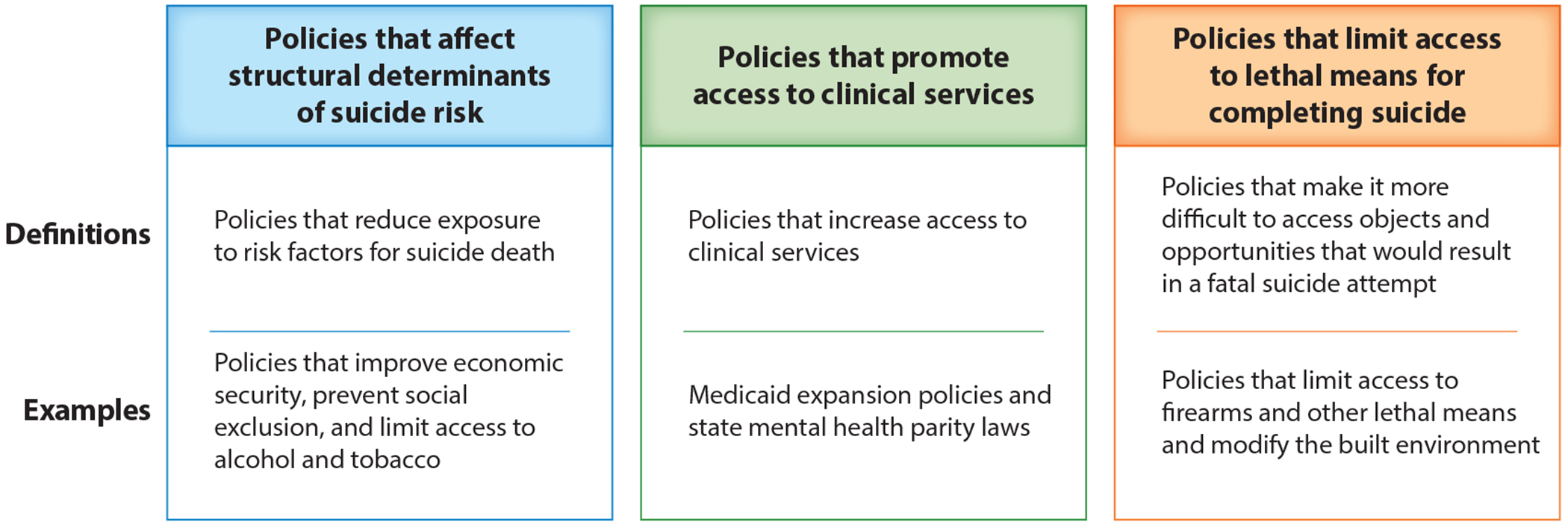
Typology of public policies to prevent suicide death.

Figure 2 offers a typology of three broad domains of public policies with demonstrated potential to prevent suicide. These domains and their definitions were informed by reviews on suicide epidemiology (6, 77, 141), suicide prevention interventions (53, 73, 80, 98, 126, 143), and population-based approaches to mental health (102). The domains are not mutually exclusive, and the typology is intended not to provide rigid categories but rather to offer structure to inform research and policymaking related to suicide prevention. The text below summarizes evidence about policies in these domains. Of note, most of the policies reviewed—with the exception of some of those related to lethal means restriction—are not adopted with the explicit goal of preventing suicide. Rather, suicide deaths being averted is an unintended benefit of economic, social, and regulatory policies that were adopted to achieve other goals (e.g., improve economic security, prevent chronic disease, increase access to health care).

### Policies that Affect Structural Determinants of Suicide Risk

Public policies shape the structure of societies and, thus, risk factors for suicide death at the population level (24, 35, 42, 73, 76, 126, 127, 133). Our review identified three specific types of policies that affect structural determinants of suicide death and that have been the focus of rigorous suicide research: (*a*) policies that improve economic security, (*b*) policies that prevent social exclusion and marginalization, and (*c*) policies that limit access to alcohol and tobacco.

#### Policies that improve economic security.

Economic insecurity is a major, often chronic stressor that increases the risk of suicide death. As such, policies that improve economic security have demonstrated evidence of preventing suicide death.

##### Minimum wage laws.

Multiple studies suggest that minimum wage laws that increase the minimum wage rate prevent suicide death (37, 44, 59), though some studies report null effects (140). For example, a pooled time-series analysis of 2006–2016 data from 50 states found that a $1.00 inflation-adjusted increase in the minimum wage was independently associated with a 1.9% decrease in the suicide death rate, with minimal effect heterogeneity by race and sex (44). These minimum wage increases translated into an estimated 8,000 suicides prevented between 2006 and 2016.

Studies have also found that the protective effects of minimum wage on suicide death are concentrated largely among individuals with high school or less as their highest level of educational attainment. This finding strengthens confidence in a causal relationship because these are individuals with lower average incomes for whom the policy would thus presumably have the strongest effects. For example, Kaufman and colleagues (59) found that a $1.00 inflation-adjusted increase in the minimum wage resulted in a 3.4%–5.4% decrease in the suicide rate among nonelderly adults with a high school education or less. State unemployment rates modified this relationship, with the largest inverse effects at higher unemployment levels. No effect was observed for nonelderly adults with a college education or more. Dow and colleagues (37) found similar results, with effects being largest among adults with a high school education or less.

##### Unemployment benefits.

Research on the effects of unemployment benefit policies on suicide is limited, but existing literature suggests a protective effect (32, 59). For example, Cylus and colleagues (32) analyzed state unemployment benefits, unemployment rates, and suicide data between 1968 and 2008 and found that more generous unemployment benefit policies weakened the strength of the positive association between unemployment rates and suicide death rates, with similar results across age and sex categories.

##### Paid sick leave.

The health and social impacts of paid sick leave policy generosity have been the focus of extensive research, generally demonstrating positive effects, but little work has focused on outcomes related to suicide (85). Wolf and colleagues’ (140) pooled time-series analysis of 2006–2016 data from 50 states found that each one-hour increase in paid sick leave requirement was independently associated with a 0.1% decrease in the male suicide death rate but was not significantly associated with the female suicide death rate.

##### Supplemental Nutrition Assistance Program.

Two studies suggest that policies that increase Supplemental Nutrition Assistance Program (SNAP) participation may prevent suicide. Rambotti (109) pooled state-level data on SNAP participation, suicide death, and a range of covariates from 2000 to 2015 and found that a one standard deviation increase in SNAP participation (4.5% of a state’s population) resulted in an estimated 31,612 suicides prevented during the study period. Austin and colleagues (4) found that the combination of two state policies that increase SNAP enrollment—eliminating asset testing (i.e., not considering household assets as an eligibility criterion) and increasing the income limit—were associated with a reduction in suicide death rates. Of note, unintentional motor vehicle death rates were used as a nonequivalent dependent variable and were found not to be associated with SNAP policies (52).

#### Policies that prevent social exclusion: policies that prohibit discrimination on the basis of sexual and gender identity.

An increasing number of states have adopted policies that prohibit discrimination on the basis of a person’s sexual or gender identity. Structural stigma theory (52) suggests that these policies may prevent suicide death among sexual and gender minorities (118), but a major challenge to documenting such effects is the absence of comprehensive and systematic data about sexual orientation and gender identity in the National Violent Death Reporting System (26). Given this limitation, we review studies of the effects of state policies on nonfatal suicide-related thoughts and behaviors.

Prairie and colleagues (99) used repeated cross-sectional data from the Youth Risk Behavior Survey (YRBS) and found that enumeration of sexual orientation as a protected class in state hate crime laws was associated with a 1.2-percentage-point decrease in self-reported suicide attempts among all high school students, corresponding to a 16.1% relative reduction in attempts. A significant, positive interaction was observed between these laws and the respondent identifying as “bisexual” or “questioning” compared with “gay” or “lesbian.” Cunningham and colleagues (30) found a positive correlation between a state passing an antitransgender rights bill and Internet searches related to suicide, with effects that were stronger in states with larger lesbian, gay, bisexual, and transgender populations. Raifman and colleagues (107) analyzed YRBS data from 1999–2015 and used a difference-in-differences approach to examine the effect of same-sex marriage laws on rates of self-reported suicide attempts among high school students. The analysis found that a law’s passage was associated with a 7% relative reduction in self-reported suicide attempts, with effects stronger among youth who identified as sexual minorities.

#### Policies that limit access to alcohol and tobacco.

Individual-level interventions that reduce alcohol consumption reduce suicidal ideation and behaviors (139); and well-designed epidemiological studies (e.g., prospective cohort and twin studies) indicate that smoking incidence and intensity are positively and causally associated with the risk of suicide death (66, 67). Consistent with these findings, policies that limited access to and use of alcohol and tobacco have demonstrated evidence of preventing suicide death.

##### Alcohol-related policies.

Multiple systematic reviews indicate that policies that restrict access to alcohol prevent suicide death (45, 64, 142). Before 1988, when federal law began to require states to have a minimum legal drinking age (MLDA) of 21 in order to receive federal highway funds, there were time-varying MLDA policies between states. This variation provided opportunities for natural experimentation (135). Overall, there is evidence of MLDA laws preventing suicide death (11, 57, 142). Grucza and colleagues (48) found that increasing the MLDA to 21 has had enduring effects and prevents an estimated 600 suicides annually. Carpenter & Dobkin (20) used a regression discontinuity design to examine the effect of turning 21 (the national MLDA) on suicide death rates among adults ages 19–22 in the United States between 1997 and 2004 and found that turning 21 was independently associated with a 16% increase in suicide mortality.

There is wide between-state variation in alcohol tax policies. Multiple studies suggest that policies that increase alcohol tax rates may decrease suicide death rates, although some studies offer conflicting evidence. In a meta-analysis, 5 of the 12 effects identified found a significant inverse association between alcohol prices and taxes and suicide death rates (134). A study using data from 1976–1999 concluded that a 5.5-cent increase in beer tax would prevent about one male between the ages of 15 and 19 from dying by suicide each year as well as one male between the ages of 20 and 24 but would have no effect on female suicide death rates (75). Son & Topyan (125) used state-level data from 1995–2004 and found that wine excise taxes reduced suicide death rates among adults ages 25–64 but that beer and spirit taxes had no effect on suicides.

All states currently have some form of zero tolerance law, but states adopted these laws at different times and laws vary in terms of the specific blood alcohol concentration level that is considered zero. Some evidence indicates that these policies prevent suicide. Carpenter (19) analyzed the data on zero tolerance laws and suicide between 1981 and 1998 and concluded that the laws reduced suicides by 10.3% among males ages 15–17 and by 7.7% among males ages 18–20 but had no effect on older males or females. Similarly, Markowitz and colleagues (75) found that zero tolerance laws were inversely associated with male and female suicide death rates among those ages 15–19.

##### Tobacco-related policies.

Some evidence demonstrates that policies that reduce smoking rates prevent suicide death. In a study of variations in cigarette excise taxes and smoke-free air policies across states and suicide death rates between 1990 and 2004, Grucza and colleagues (50) found that a $1 increase in cigarette excise tax was independently associated with 11% lower odds of suicide death [adjusted odds ratio: 0.89, 95% confidence interval (CI): 0.87, 0.93] and that a one-point increase in smoke-free air policy score (on a six-point scale) was independently associated with 2% lower odds of suicide death. The magnitudes of effect sizes were larger in demographic groups with higher smoking rates, strengthening confidence in a causal association. An interrupted time-series study of tobacco packaging and pricing policies in South Korea also found evidence of these policies reducing suicide mortality (56).

### Policies that Promote Access to Clinical Services

Individually focused clinical interventions have demonstrated effectiveness in preventing suicide (53, 73, 80, 98, 143). Policies that promote access to clinical services have the potential to reduce suicide death rates in at least three ways. First, these policies may enhance direct access to suicide-specific interventions via improved insurance coverage. Second, these policies may enhance access to suicide-specific interventions as well as other protective mental health services via greater exposure to suicide risk screenings in health care settings. Third, these policies could contribute to the prevention of suicide by reducing suffering caused by needing to forgo necessary medical care.

#### Medicaid expansion.

Numerous studies suggest that Medicaid expansions under the Affordable Care Act are associated with reductions in suicide death (5, 8, 12, 91, 92), although at least one study identified a null effect (71). Austin and colleagues (5) used 2005–2017 suicide data from 8 Medicaid expansion and seven nonexpansion states and found that expansion was associated with 1.2 fewer suicide deaths per 100,000 population annually. Patel and colleagues (92) used a difference-in-differences design and concluded that Medicaid expansion across the United States resulted in an estimated 1,818 suicides prevented between 2015 and 2018 (5, 8, 12, 91). Ortega (91) found that Medicaid expansion was independently associated with increases in admissions to mental health treatment facilities, increases in Medicaid-reimbursed prescriptions for medications used to treat common mental disorders, and modest decreases in suicide death rates. Among adults ages 18–39 with cancer—a population at elevated risk for suicide (55)—Barnes and colleagues (8) found that Medicaid expansion was independently associated with a lower suicide death rate.

#### State mental health parity laws.

To complement federal parity laws, states have gradually adopted their own mental health parity laws, and difference-in-differences studies indicate that these laws are independently associated with reductions in suicide death rates (41, 65, 124). For example, Lang (65) found that between 1990–2004 state adoption of a mental health parity law was associated with an approximate 4% reduction in the state suicide death rate the following year. Solomon & Dasgupta (124) analyzed 1998–2008 data and concluded that state parity laws decreased the suicide rate by 3.1% among the college-age population.

### Policies that Limit Access to Lethal Means for Completing Suicide

Policies in this domain generally aim to make it more difficult to access means that would result in a fatal suicide attempt. Population subgroups are often targeted, such as child-access prevention (CAP) laws and laws that require a minimum age to purchase or possess firearms. This targeting of specific population subgroups provides researchers the opportunity to assess the impact of these laws using negative control analyses (e.g., nonequivalent dependent variables) in addition to other forms of hypothesis testing (130). Reducing access to lethal means as a suicide prevention approach rests on several well-established observations: First, there is wide variation in the lethality of different means used in suicide attempts (17); second, the method used in suicidal acts is determined largely by what is readily at hand; and third, impulsivity often plays a role in suicidal behavior (116). A key methodological consideration when assessing evidence on policies related to lethal means restriction is substitution effects (34, 81).

#### Policies that limit access to firearms.

Suicide attempts involving a firearm have the highest fatality rate at ~90%, compared with ~56% for drowning and 53% for hanging (27). More than half (53%) of suicides in the United States in 2020 involved a firearm, and there is wide between-state variation in firearm suicide death rates (117). For example, in 2020, the per-100,000 firearm suicide death rate ranged from 1.8 in New Jersey and Massachusetts to 20.9 in Wyoming (absolute difference = 19.1), whereas the nonfirearm suicide death rate ranged from only 4.6 in Mississippi to 11.4 in South Dakota (absolute difference=6.8). This between-state variation in firearm suicide death rates is likely influenced by differences in firearm ownership rates. Because the stringency of firearm laws is, in general, inversely related to the prevalence of firearms (e.g., Massachusetts has more stringent laws than Montana), cross-sectional assessments of the effects of firearm policies are problematic because ownership prevalence confounds the stringency of laws. In the summary that follows, we follow RAND in considering only prepost studies.

Strong epidemiological evidence demonstrates that having a firearm in the home increases the risk of suicide death (3, 60, 72, 73, 110, 129). For example, data from the LongSHOT project—a longitudinal dataset with individual-level administrative data on handgun ownership among ~28.8 million California adults between 2004 and 2016 (145)—indicate that becoming a handgun owner significantly increases the risk of firearm suicide (hazard ratio = 7.82 for men and 35.15 for women) (129) and that when a woman lives with someone who becomes a handgun owner, the woman’s risk of dying by suicide significantly increases—by ~50% (attributable to an increase in firearm suicide by more than 400%) (83). Furthermore, the bias-adjusted estimate of the extent to which the risk of dying by suicide decreases after divesting of firearms is approximately as large as the increase in risk associated with becoming a handgun owner (131). Evidence about the effects of firearm policies on suicide death rates, however, is more equivocal.

In 2023, RAND published a comprehensive systematic review of the effects of 18 types of firearm policies on a range of outcomes (123). In terms of preventing suicide death, the review concluded that there was supportive evidence for one of these policies, moderate evidence for two of these policies, and limited or inconclusive evidence for nine of these policies and that no studies had evaluated six of these policies. Based on the results of three qualifying studies (62, 119, 137), CAP laws were deemed to have moderate evidence of preventing total suicides, regardless of means, among young people ages 14–20 and inconclusive evidence of reducing total suicides in the larger population. Based on results of five qualifying studies (2, 58, 108, 112, 137), laws that set 21 as the minimum age of firearm purchase were deemed to have moderate evidence of preventing firearm suicides among young people and inconclusive evidence that policies that establish higher minimum age requirements affect total suicides. Based on results of two qualifying studies (38, 70), policies that establish waiting-period requirements for a firearm purchase have moderate evidence of preventing firearm suicides in the total population and limited evidence of affecting total suicides.

#### Policies that modify the built environment.

Policies that fund the construction of barriers and other modifications to built environments to prevent suicides at hot spots (e.g., high bridges) demonstrate fairly strong and consistent effects (28, 89, 93, 95, 96), with some exceptions (122), and are considered cost-effective (7). For example, a Cochrane review of 13 prepost studies that evaluated the effects of modifications to built environments (e.g., erecting barriers on bridges) found that rates of suicide death by jumping decreased from 5.5 to 0.8 suicides per year (89). Although the studies often observe some substitution effects, a net reduction in suicides is still typically observed. For example, a meta-analysis of built environment modifications to prevent jumping suicides at hot spots observed an 86% reduction in jumping suicides per year at the hot spots, while there was a 44% increase in jumping suicides per year at nearby sites. However, the net benefit was a 28% reduction in all jumping suicides per year in cities included in the analysis (95% CI = 13%, 40%) (96). Policies that place closed-circuit television and video devices at suicide hot spots also demonstrate the potential to prevent suicide (90, 115).

#### Policies that limit access to poisoning means.

Policies that make it more difficult to access fatal amounts of poison have demonstrated evidence to prevent suicide death via these means, with little evidence of substitution effects. Lim and colleagues (68) published a systematic review of 62 evaluations from 26 countries and found that policies that restricted access to poisoning means were associated with reductions in suicide by these means. For example, the median interquartile range (IQR) of the prepost policy change in poisoning-specific suicide rate was −1.18 (−2.03 to −0.46) per 100,000 people, while the median IQR change in overall suicide rate, regardless of means, was −0.09 (−2.22 to +1.65). Policies that have restricted specific poisoning means include detoxification of domestic gas (16), mandatory use of catalytic converters in motor vehicles (113), and restrictions on access to pesticides (51) as well as medicines such as barbiturates and analgesics (e.g., removing drugs with high toxicity from the market, shifting from over-the-counter to prescription-required access, limiting pack size).

## KEY CONSIDERATIONS FOR POLICY IMPLEMENTATION IN SUICIDE PREVENTION

**Figure 3:**
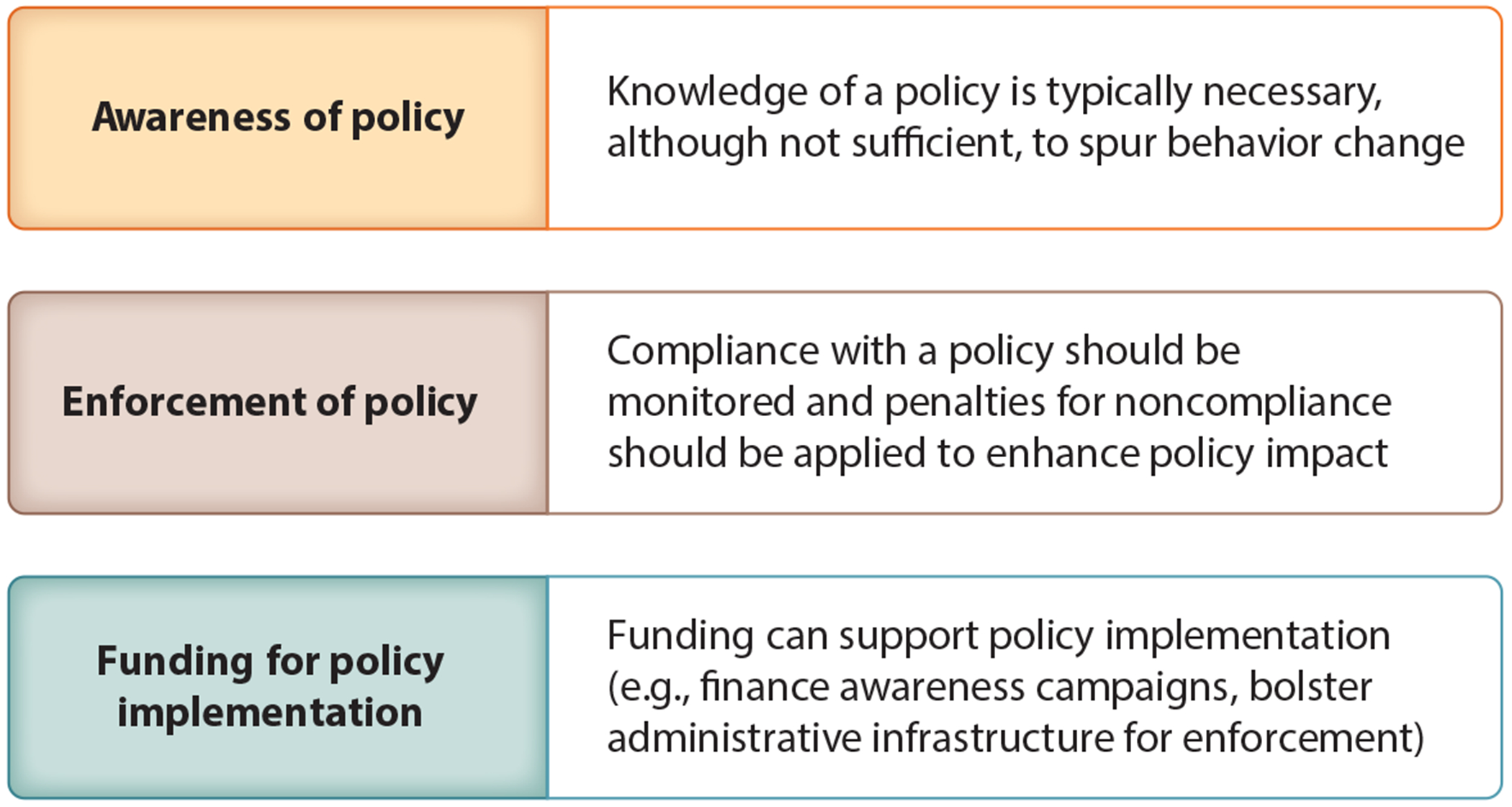
Domains to consider in policy implementation.

Policies on the books (i.e., codified in the text of laws and regulations) generally have little impact if key populations are not adequately aware of the policies, if policies are inadequately enforced, and if sufficient funding is not allocated for policy implementation (69). Awareness, enforcement, and funding are three domains of variables that are—to varying degrees, depending on the specifics of the policy—essential to policy implementation success (Figure 3) (101). There is value in studying the policy implementation processes that mediate relationships between policies and suicide outcomes because doing so, as Burris and colleagues (14) describe, “opens the black box in which the operation of policies and the practical factors that influence enforcement and compliance often hide” (p. 2). However, policy implementation variables of awareness, enforcement, and funding have received sparse attention in policy research related to suicide (79). Conceptual frameworks—such as the integrated framework of policy implementation (13); the theory of street-level bureaucracy (69); and the exploration, preparation, implementation, and sustainment framework (29)—can inform research and practice to understand and improve the implementation and effects of policies on preventing suicide death.

For example, consider CAP laws. A presumed mechanism through which these policies prevent suicide is by adults becoming motivated to store firearms more safely, and in turn engage in safe storage behaviors, because they fear criminal penalties for noncompliance. A precondition for this mechanism to be activated and for safer storage to occur is awareness about the CAP law. To enhance the implementation and effects of CAP laws, a state agency could launch an informational campaign about the CAP law, penalties for noncompliance, and recommendations for safe storage. To study the extent and mechanisms of policy implementation, public opinion surveys—ideally of samples of firearm owners with children in the home—could be used to assess awareness of the policy and the extent to which it has prompted safer storage behaviors.

An example related to enforcement, a presumed mechanism through which state mental health parity laws prevent suicide is by insurance companies offering more generous coverage for mental health services in their plans. If there is insufficient enforcement of parity laws—which relates to sufficient funding for administrative infrastructure to support implementation—and only marginal penalties for parity violations, insurance companies are unlikely to be motivated to alter their plan offerings. Some prior work has demonstrated weak enforcement of parity laws (39, 100), suggesting that the effects of state parity laws on preventing suicide death may be larger if parity policy enforcement was stronger. Understanding the mediating effects of policy implementation processes on suicide-related policy outcomes is an important area for future research.

## PRIORITY AREAS FOR FUTURE RESEARCH ON POLICY AND SUICIDE

### Policies Related to Social Media and Internet Regulation

Social media and the Internet have become dominant sources for information, and misinformation, about suicide and lethal means. Analyses of social media data find significant presence of graphic images and videos related to suicide (9, 33); in addition, chat forums specifically for suicidal individuals often normalize and romanticize suicide and function as a venue for sharing information about lethal means (18, 54, 87, 146). At the same time, however, social media and chat forums are often places where suicidal individuals seek help and connection to reduce suicidal crises, and youth report using social media and discussion forums for social support when distressed and to identify crisis support (74). Thus, developing public policies that prevent exposure to harmful suicide-related content online, while simultaneously promoting positive interactions and connections to crisis support, is an important area for future research.

Existing efforts to reduce exposure to harmful suicide content online are driven predominately by individual platform content moderation. Major Internet and social media platforms (e.g., Google, Meta, X, TikTok) have policies to flag and remove content that promotes self-harm and provide users with resources (e.g., crisis help information) when search terms indicate the potential for a suicidal crisis. However, these policies are insufficient to remove all content, and many forums where suicide content is discussed are unmoderated.

Guidelines to promote safe discussion of suicide online are being developed, which should be studied and could serve as a basis for public policies (111). Other priority areas for policy-relevant research on social media, Internet use, and suicide relate to (*a*) methods to identify groups of individuals who are particularly vulnerable to exposure to inappropriate suicide content online, (*b*) the establishment of guidelines for reporting about suicide online (88), and (*c*) studies that assess the effects of online awareness campaigns and help-seeking interventions (94).

### Research on Policies Related to Suicide Crisis Lines

Suicide crisis lines—such as the National Suicide Prevention Lifeline, now the 988 Suicide and Crisis Lifeline in the United States—are a core component of suicide prevention strategies around the world and are supported by public policies (e.g., policies related to funding and quality standards). Some evidence indicates that these crisis lines prevent suicide (86). Thus, policies that increase the use of suicide crisis lines and the quality of services provided may prevent suicide deaths. The policy landscape related to crisis lines in the United States is rapidly evolving as states are adopting different policy approaches to fund 988 implementation (105), and future research should explore the effects of these policies on suicide death rates.

### Research on Cannabis and Opioid Policies

Numerous studies have assessed the effects of medical and recreational cannabis laws on suicide rates, generally finding null effects (1, 36, 78, 114). However, there is some evidence of effect heterogeneity, with these policies potentially increasing suicide rates among young males (1, 36, 78), although this finding is not consistency observed (49). In terms of opioids, one study assessed the effect of Florida’s prescription drug monitoring program and found a null effect on suicide death rates (40). Cannabis and opioid policies warrant future research as cannabis markets mature and more time elapses since the initial implementation of new cannabis and opioid policies.

### Research on Reproductive Care Policies

State policy landscapes related to reproductive care are rapidly changing in the United States. One well-designed study has assessed the effects of policies that limit access to reproductive care on suicide death. Using a difference-in-differences design and data from 1974–2016, Zandberg and colleagues (144) found that policies targeting the regulation of abortion providers were independently associated with a 5.8% higher suicide rate among reproductive-age women (ages 20–34) but not postreproductive-age women (ages 45–64) nor motor vehicle death rates (which served as a nonequivalent dependent variable). The study results offer evidence that policies that impose barriers to reproductive care may causally affect suicide death rates, but more research is needed.

### Research on Suicide Policy Communication

The extent to which evidence about policies to prevent suicide saves lives hinges on the degree to which it is translated into policies that are widely adopted and rigorously implemented. Evidence is just one of many factors, however, that influence policymaking and implementation decisions (15). To enhance the influence of evidence on policymaking and implementation, policy-focused dissemination research on issues such as child development and chronic disease has used experimental designs to generate knowledge about how to communicate evidence so that it resonates with policymakers, is engaged with, is cognitively processed, and is used in decision-making (103, 121). Such research has also identified unintended consequences of communication and identified ways not to frame evidence for certain policymakers (104, 138). No such policy-focused dissemination studies to our knowledge, however, have focused on suicide specifically. Such research may have particular utility for the issue of suicide because many of the policies identified in this review are politically contentious (e.g., policies that increase the minimum wage, policies the restrict access to lethal means).

## CONCLUSIONS

Many well-designed quasi-experimental studies provide evidence that public policies can prevent suicide death. Most of these policies are not focused explicitly on suicide, and suicide deaths prevented are an unintended benefit of policies that are adopted to achieve other goals. This notion highlights the importance of considering social determinants in suicide prevention. Policies related to improving economic security, limiting access to alcohol, and restricting access to lethal means demonstrate the strongest evidence of preventing suicide death in the United States. Future research and practice should place greater emphasis on understanding and advancing policy implementation processes that may mediate relationships between policies on the books and suicide deaths in society.

## INCREASING CONCERN ABOUT SUICIDE AMONG POLICYMAKERS IN THE UNITED STATES

A search for “suicide” in Congress.gov—a comprehensive database of bills introduced in the US Congress—reveals that the first bill that mentioned “suicide” was introduced in 1955 (H.J.Res.256). The bill became law (Pub. L. 84-182) and identified suicide as a consequence of untreated mental illness, and it explicitly authorized the US Surgeon General to fund mental health research. In state legislatures, where most public health policy authority sits in the United States (47), suicide has received increasing policy attention. As shown in Figure 1, the number of new state laws mentioning suicide increased dramatically between 1993 and 2023. In 2022, 334 state laws were passed that mentioned suicide, compared with 77 in 2003. Suicide has also received increasing attention from state legislators on social media—an indicator of the prominence of suicide on their policy agendas (106). Only 3.2 of every 10,000 social media posts from state legislators mentioned suicide in 2013 compared with 26.4 in 2019 and 19.6 in 2023.

## SUMMARY POINTS

1. Many well-designed studies provide evidence that public policies can prevent suicide death.
2. Economic, social, and regulatory policies that are not explicitly focused on suicide can prevent suicide.
3. Policies that improve economic security, limit access to alcohol, and restrict access to lethal means demonstrate the strongest evidence for preventing suicide death.
4. Future research and practice should attend to policy implementation processes because they may mediate relationships between policies and suicide death outcomes.

## Abbreviations

Lethal means: objects (e.g., firearms, poisons) and opportunities (e.g., high-elevation sites without barriers) that increase the likelihood of a suicide attempt being fatal
Policy effect heterogeneity: the extent to which the magnitude and direction of a policy’s effect vary across different population groups
Minimum wage laws: establish the lowest amount an employer can pay an employee per hour
Unemployment benefits: temporary replacement of a portion of wages for workers who have lost their jobs, as long as they continue looking for work
Paid sick leave: short-term, paid time off that a worker can use when they or a loved one is sick, injured, or receiving medical treatment
Supplemental Nutrition Assistance Program (SNAP): provides individuals and families with incomes below a threshold with an electronic benefit transfer card to buy groceries at authorized food stores and retailers
Nonequivalent dependent variable: a variable that would plausibly be affected by factors that would confound an observed association between an independent and dependent variable but would not be affected by the independent variable
Zero tolerance laws: make it a criminal offense for motorists under age 21 to have any alcohol in their system while driving
Medicaid: a joint federal–state health insurance program that provides health insurance to eligible groups, primarily those with incomes below a threshold
Difference-in-differences design: a quasi-experimental design in which the magnitudes of within-state, prepost changes in the dependent variable are compared between states that did versus did not adopt a specific policy
Child-access prevention (CAP) laws: allow for the prosecution of adults who intentionally or carelessly allow children to have unsupervised access to firearms
Substitution effects: the extent to which suicide rates via other means increase following implementation of a policy that restricts access to a specific lethal means
State mental health parity laws: statutory requirements for health insurance companies to provide equal benefit coverage for mental health and medical/surgical services
